# Relationship Between Childhood Sensory Processing Differences and Quality of Life as Adults

**DOI:** 10.3389/fpsyg.2022.886833

**Published:** 2022-07-27

**Authors:** Teresa A. May-Benson, Alison Teasdale, Olivia Easterbrooks-Dick

**Affiliations:** ^1^TMB Educational Enterprises, LLC, Norristown, PA, United States; ^2^SPIRAL Foundation, Newton, MA, United States; ^3^OTA The Koomar Center, Newton, MA, United States

**Keywords:** sensory processing, quality of life, longitudinal, motor coordination, physical health

## Abstract

Research suggests a relationship between sensory processing, motor skills and quality of life (QOL) in a variety of clinical populations of adults and children. There have been no studies which investigated the relationship of childhood sensory processing and integration and related motor performance (sensori-motor) patterns identified using an Ayres Sensory Integration® (ASI) frame of reference and later QOL of those children as adults. This longitudinal follow-up study examined this relationship. Adult QOL was also examined in relation to current adult sensori-motor patterns. Fifty-three adults who received occupational therapy services as children, were identified as having sensori-motor difficulties at that time and completed a sensory history and a quality-of-life measure as adults participated. Measures included the *OTA the Koomar Center Sensory History (SXHX*), *Adult/Adolescent Sensory History (ASH), and the World Health Organization Quality of Life-Brief* (*WHOQOL-BREF*). MANCOVA found that Total childhood sensori-motor scores had a small relationship to Physical Health QOL as adults that approached significance. Pearson Correlations found that adults with childhood sensori-motor challenges who report sensori-motor challenges as adults had a moderate significant relationship among overall sensori-motor functioning and Physical Health (*r* = −0.56, *p* = 0.018). Visual (*r* = −0.76, *p* = 0.001), movement (vestibular; *r* = −0.48, *p* = 0.042) and tactile processing (*r* = −0.63, *p* = 0.008) had moderate to large significant relationships with Physical Health. Visual processing (*r = −0.54, p = 0.024*) was also significantly related to Psychological Health. Motor Coordination trended to significance for Physical Health (*r* = −0.42) and Psychological Health (*r* = −0.41). Conversely, adults who reported typical sensori-motor scores as adults, despite childhood sensori-motor challenges, had a good QOL. Furthermore, similar to previous research, there was a relationship between current visual, movement (vestibular) and tactile (touch) sensory processing and adult Physical and Psychological Health. A multivariate linear regression found Sensory Discrimination and Modulation accounted for one-quarter of the variance in QOL in adults with only Discrimination being statistically significant. Therefore, it is important to consider childhood sensori-motor function as well as adult functioning when examining QOL. Further, heretofore unexamined Sensory Discrimination was found to play a role in adult QOL.

## Introduction

Occupational therapists using an Ayres Sensory Integration® (ASI) frame of reference who work with children experiencing sensory processing and integration challenges typically treat children with a variety of diagnoses including ADHD, Autism Spectrum Disorder, trauma and attachment disorder and mental health conditions (Bundy and Lane, 2020). However, ASI intervention was originally designed to address sensory processing and integration problems with related motor performance challenges, such as postural control and praxis challenges, (Ayres sensori-motor patterns) in children with learning disabilities and those with no other diagnoses (Ayres, 1972). Application of this model of intervention rapidly spread to use with other diagnoses and in recent years the majority of research on ASI has been focused on children with ASD and the population of children with Ayres sensori-motor challenges who have no other diagnoses has been largely ignored. Particularly with this population of children without other diagnoses, clinicians report that parents question how these sensori-motor challenges may impact the long-term health, well-being and social participation success of their children (Cohn et al., 2000). Thus, an important aspect of evidence-based practice is examination of the prognosis of a given disorder or difficulty on later performance of functional skills, health and well-being. There is little to no such prognosis information available in any area of functioning regarding children with Ayres sensori-motor challenges, particularly those with no other diagnoses.

A few studies have examined the longitudinal outcomes of various sensory processing and integration patterns in children over the course of relatively short periods of time extending approximately 2–6 years (McCormick et al., 2016; Perez Repetto et al., 2017; Baranek et al., 2019). No studies, however, have followed children with known sensory processing and integration challenges (and related postural and motor challenges) into adulthood to examine potential long-term implications of these childhood sensori-motor challenges. The authors of this paper have endeavored to follow-up with children with known Ayres sensori-motor challenges who had no other known diagnoses to examine various aspects of adult functioning in these individuals over a period of five to 25 years. Sensori-motor functioning, diagnoses, education, employment, and quality of life (QOL) were areas of particular interest as they are areas of long-term concern frequently expressed by parents in clinical practice. Detailed results of this follow-up study regarding changes in sensori-motor functioning from childhood to adulthood and relation to education and employment will be presented elsewhere. This paper will examine the relationship of childhood patterns of Ayres sensori-motor challenges and adult sensori-motor characteristics as they relate to adult QOL. The authors sought to determine if early sensori-motor characteristics were related to or could predict adult QOL and to examine what adult QOL is like in adults who have known childhood Ayres sensori-motor challenges whether those sensori-motor characteristics are still present in adulthood or not. In this paper, the terms sensori-motor patterns and sensori-motor characteristics will refer specifically to sensory processing and integration (and related postural and motor skills) characteristics and patterns identified using an ASI theoretical model and will inclusively refer to individuals with patterns of sensory modulation, sensory discrimination, and postural control/motor performance/praxis function.^1^ Further, although there continues to be some debate about terminology regarding motor performance patterns referred to variously as dyspraxia, praxis challenges or developmental coordination disorder (DCD), recommendations by Gibbs et al. (2007) suggest that examination of literature across these diagnostic labels can be useful.

## Literature Review

Quality of life (QOL) is a concept that has been identified by the World Health Organization to approach health from a holistic lens, which considers the impact of disease or impairment on areas such as daily activities, behavior, and perceived health (World Health Organization, 1996). It is considered a primary indicator of overall health and wellness. The World Health Organization (WHO) defines QOL as “an individual’s perception of their position in life in the context of the culture and value systems in which they live and in relation to their goals, expectations, standards and concerns” (World Health Organization, 2022). Aspects of QOL include current physical and psychological health, social relationships, and environmental components (World Health Organization, 1996). Further, QOL has been defined in different ways and may be influenced by numerous factors across the lifespan including medical diagnoses, cardiorespiratory and muscular fitness status, social support and participation (Tal-Saban et al., 2014; Engel-Yeger et al., 2016; Wallander and Koot, 2016; Lin and Huang, 2019; Jovellar-Isiegas et al., 2020; Bermejo-Cantarero et al., 2021; Costa-López et al., 2021; Lestari et al., 2021).

Studies examining sensory processing and integration differences (including related praxis and motor challenges) as defined by a number of theoretical frameworks (e.g., Ayres Sensory Integration®, Dunn’s Model of Sensory Processing, developmental coordination disorder) have identified sensory and motor factors that may impact QOL in both children and adults (Kinnealey et al., 2011; Lin and Huang, 2019; Costa-López et al., 2021). Costa-López et al. (2021) conducted a systematic review of 14 studies examining sensory processing and QOL. All but one study examined adults and the remaining article examined adolescents. While studies which examined QOL in children and adolescents have been conducted, these have exclusively examined children and adolescents with various physical disabilities or medical conditions such as cerebral palsy (Makris et al., 2021), asthma (Kouzegaran et al., 2018), traumatic brain injury (Di Battista et al., 2012), disease conditions (Wolf et al., 2018; Wardoku et al., 2019; Marschner et al., 2020) and trauma and mental health challenges (Bastiaansen et al., 2020; Lawson et al., 2020; Pinto et al., 2021). A number of studies have examined QOL in children with autism with or without specifically identified sensory processing challenges (Benen Demchick et al., 2014; Chuang et al., 2014; Xu et al., 2019; Leader et al., 2021; Oakley et al., 2021). However, to date no studies have been located which examine QOL in children with only Ayres sensori-motor challenges and none have examined the relationship between these early sensori-motor challenges and later adult QOL.

Research on sensory processing and QOL has been conducted primarily on those groups with sensory modulation or sensory-over-responsivity patterns largely using Dunn’s (1999) or Miller et al. (2007) models of sensory processing. No studies were found that examined sensory discrimination patterns in relation to QOL in either children or adults. Studies have been conducted on the relationship of various patterns of sensory processing to QOL in groups with and without additional medical diagnoses, including mental health diagnoses such as bipolar disorder (Engel-Yeger et al., 2016); autism (Lin and Huang, 2019); and cerebral palsy (Jovellar-Isiegas et al., 2020). Results found some similarities and differences in the sensory processing patterns of these different groups and their patterns of QOL. Lin and Huang (2019) found that autistic adults with sensory processing challenges scored significantly lower on QOL measures than their neurotypical counterparts. These autistic adults scored higher (more dysfunctional) on the four sensory processing quadrants defined by Dunn than the neurotypical group and had higher levels of anxiety (Lin and Huang, 2019). High sensory sensitivity was also related to decreased performance in both the Physical and Psychological Health domains of QOL for this group of autistic adults (Lin and Huang, 2019). Further, Kinnealey et al. (2011) investigated the relationship of sensory modulation and health-related quality of life (HRQOL) in adults without clinically diagnosed mental health conditions or confounding medical diagnoses and found that three out of four sensory processing patterns (sensory sensitivity, sensory avoiding, and low registration) were correlated with role emotional and mental health factors of HRQOL. In this study, a sensory over-responsive group had similar HRQOL as a non-sensory-overresponsive group in the areas of “physical functioning, role physical, role emotional, and mental health,” however, there were significant differences in the areas of “bodily pain, general health, vitality, and social functioning” (Kinnealey et al., 2011, p. 325). In a group of college students, Hwang et al. (2021) found that individuals with low registration and sensory sensitivity were negatively correlated with QOL and resilience, except for the social domain. More recently, Costa-López et al. (2021) conducted a systematic review to better understand the relationship between sensory processing and QOL. This review found that high sensitivity to sensory stimuli may have a negative impact on QOL. Overall, these findings support a relationship between aspects of current sensory processing and QOL in adults.

Some studies examined the relationship of motor performance challenges and QOL (Caçola and Killian, 2018; Engel-Yeger, 2020). No studies were found which specifically examined praxis or motor performance challenges as identified by ASI; however, studies were located which examined DCD and QOL. These studies can inform this topic as praxis, as conceptualized within ASI theory, is viewed by many as a subtype of DCD (Vaivre-Douret et al., 2016) and Gibbs et al. (2007) suggest that labels of DCD and dyspraxia essentially identify the same children. This view was further supported by Cermak and May-Benson (2020). In addition, Allen and Casey (2017), Chung (2018), Delgado-Lobete et al. (2020), and Mikami et al. (2021) found relationships between sensory processing difficulties and individuals with DCD, supporting the presence of sensory processing and integration difficulties in individuals with motor coordination challenges. The relationship of motor performance challenges and aspects of QOL was noted as children with DCD were found to have lower HRQOL (Redondo-Tébar et al., 2021) and leisure activity levels (Raz-Silbiger et al., 2015) than their typically developing counterparts. Young adults with DCD also scored lower than a control group on QOL, overall participation, and life satisfaction, with the Psychological Health domain of the *WHOQOL-BREF* being a significant predictor of life satisfaction (Tal-Saban et al., 2014). Another study of adults with DCD revealed that although current levels of motor coordination did not predict QOL in this population, three behaviors potentially related to praxis challenges (avoidance, work/school impairment, social impairment) demonstrated by this group did predict QOL scores (Forde and Smyth, 2021). Further, Engel-Yeger (2020) suggested that early motor skills performance impacted later QOL. Thus, these findings suggest that sensori-motor challenges may impact aspects of QOL in both children and adults.

Much of the available research investigating relationships between various sensori-motor patterns and QOL in individuals without additional clinical diagnoses examined QOL at the group’s current age, either in childhood or adulthood. To our knowledge, there are no studies that investigate the QOL of adults who were identified as having documented childhood sensori-motor challenges. In addition, much of the research available examined only the construct of sensory modulation, which was found to impact QOL. Little evidence exists examining sensory processing in general, praxis with its related features, and QOL in individuals without additional clinical diagnoses. Since we know that adults with DCD have decreased QOL related to their motor coordination challenges, it is important to investigate the relationship between all sensori-motor constructs as defined by Ayres (Bundy and Lane, 2020) and QOL in individuals with sensori-motor challenges without other diagnoses.

The purpose of this study was to examine the relationship between early childhood sensori-motor patterns and QOL in adulthood. In addition, current QOL in adulthood as related to current sensori-motor patterns was examined in this group of individuals with known sensori-motor challenges as children.

Research hypotheses were:

1. There will be a significant relationship between childhood sensori-motor characteristics and QOL in adulthood in individuals with childhood sensori-motor challenges (i.e., poorer sensori-motor functioning will be associated with poorer QOL).
2. There will be a significant relationship between adult sensori-motor characteristics and current adult QOL in individuals with childhood sensori-motor challenges (i.e., poorer sensori-motor functioning will be associated with poorer QOL).

## Materials and Methods

This study was approved by the Spiral Foundation Institutional Review Board, protocol #1039. Informed consent, including permission to access clinical records, was obtained from all adults who participated. All information was collected in accordance with the Office of Human Rights Protection and all HIPAA protections for privacy of protected personal information were observed.

### Procedures

Information for this study was obtained from a larger longitudinal follow-up study conducted by the authors on adults with known sensori-motor challenges with no other known diagnoses who received ASI-based occupational therapy services from a private occupational therapy clinic as children. For the current study, individuals’ childhood sensori-motor data (obtained at clinical intake) were matched to their current data as an adult using name and date of birth. Childhood information was provided by the individuals’ parent/guardian and was retrieved retrospectively from the children’s clinical records. Adult information was provided by the individuals themselves by completing an online survey as part of the larger longitudinal follow-up study. Sensori-motor characteristics are known at childhood clinical intake and 5–25 years later as an adult. A QOL measure is only available as an adult.

#### Recruitment for Follow-Up Study

Former clients, identified from clinical records, eligible to participate in the larger follow-up study were individuals identified with sensori-motor differences as children but whom had no other diagnoses at the time of clinical intake. All client materials were accessed and handled in accordance with HIPAA regulations. Inclusion criteria for the follow-up study were: the child was <18 years of age at time of initial services, parent and/or the adolescent completed the practice’s sensory history (which included motor and social skills) at intake, had no diagnoses except sensori-motor challenges as reported on the sensory history, was currently aged 18–50 years at time of the follow-up study, was discharged from occupational therapy services prior to 2014, and contact information could be located.

The 1,305 eligible participants were identified via a clinic database of 4,556 records of former clients. Information on the client’s name, date of birth and discharge year was accessed. Individuals were divided into 5 groups based on discharge year to ensure that recruitment efforts across time would be roughly equal. Each group consisted of a 5-year time frame. The first group included individuals who initiated services 5–10 years prior to the start of the study and proceeded to the last group which included individuals whose initial services were > 25 years prior to the study. Within each group, names were placed in a randomized order (to ensure randomized selection of potential participants) and records screened for eligibility. Records that did not meet inclusion criteria were removed. Names, addresses and phone numbers of the client’s parents were extracted from the clinical records. Only 89 individuals in the 2009–2013 group were eligible, all were invited to participate. It was not possible to locate contact information for 33 individuals.

Once parent contact information was obtained, publicly available information from the internet was used to update the child’s contact information (e.g., parent’s name and address were searched in the publicly available white pages which lists other individuals living or associated with that address). Invitations were sent to the now-adult child at the obtained address. The invitation letters came from the clinical practice who invited their previous clients to participate in a research study conducted by the SPIRAL Foundation. By the end of the recruitment phase 1,272 individuals had been invited to participate in the research. The post office returned 117 invitations with no forwarding address. *N* = 102 (response rate of 8.8% of non-returned invitations) individuals completed one or more sections of the *Adult/Adolescent Sensory History* (ASH) and therefore were eligible for inclusion in the larger follow-up study. The online survey was completed on Qualtrics, which is secure and HIPAA compliant. Participants were asked to complete an informed consent document which included permission to access their childhood clinical records, a demographics form (which included self-report of current diagnoses), the *ASH* and the *WHOQOL-BREF*.

### Participants

Participants in the current study were individuals who participated in the follow-up study of former clients of a private occupational therapy clinic specializing in Ayres Sensory Integration® services. All participants received sensory integration-based occupational therapy services (e.g., evaluation and/or intervention) at the clinic between 1983 and 2013; and fully or partially completed a follow-up survey. Inclusion criteria for this study was that the adult completed the demographics, *ASH* and *WHOQOL-BREF* portions of the follow-up survey (*N* = 62). Exclusion criteria was that the adult self-reported a current diagnosis of ASD (*n* = 9) leaving a sample size of *N* = 53.

At time of initial childhood services, the current study sample was *M* = 7.1 years, *SD* = 3.4 years of age. At follow-up the group was *M* = 26.9, *SD* = 5.5 years of age. At clinical intake individuals had no reported diagnoses other than sensori-motor difficulties. As adults, 26 individuals reported no diagnoses and 27 reported one or more conditions of ADHD, anxiety, and/or depression. For analysis purposes, this Total Group was further divided into adults who currently reported typical sensori-motor functioning (Typical Group: *n* = 26), those who currently reported mild sensori-motor challenges (Mild Difficulties Group: *n* = 13) and those who currently reported definite sensori-motor challenges (Definite Difficulties Group: *n* = 14) based on performance on the *Adult/Adolescent Sensory History* (May-Benson, 2015). See Table 1 for participant demographics. In addition, the Total Group was divided into those with self-reported diagnoses as adults and those with no reported diagnoses for some analyses.

**Table 1:** Demographics of Total adult group and *ASH* subgroups.

	Total group	Typical functioning	Mild difficulties	Definite difficulties
Gender				
Male	*n* = 32 (60%)	*n* = 9 (35%)	*n* = 5 (39%)	*n* = 7 (50%)
Female	*n* = 21 (40%)	*n* = 17 (65%)	*n* = 8 (62%)	*n* = 7 (50%)
Intake age	*m* = 7.1 years	*m* = 6.5 years	*m* = 6.8 years	*m* = 8.4 years
	*sd* = 3.4 years	*sd* = 3.5 years	*sd* = 2.8 years	*sd* = 3.2 years
	Range 1.3–16.0 years	Range 1.3–15.8 years	Range 3.2–12.9 years	Range 4.3–16.0 years
Current age	*m* = 26.9 years	*m* = 26.4 years	*m* = 27.1 years	*m* = 27.7 years
	*sd* = 5.5 years	*sd* = 4.7 years	*sd* = 6.8 years	*sd* = 5.7 years
	Range = 18–39 years	Range = 18–34 years	Range = 18–39 years	Range = 18–35 years
Education				
High school	*n* = 8 (15%) (*n* = 7^1^)	*n* = 4	*n* = 2	*n* = 2
Some college/associates	*n* = 10 (19%) (*n* = 5^1^)	*n* = 4	*n* = 5	*n* = 1
Bachelors	*n* = 20 (38%) (*n* = 4^1^)	*n* = 11	*n* = 4	*n* = 5
Post graduate	*n* = 12 (23%) (*n* = 3^1^)	*n* = 5	*n* = 2	*n* = 5
Doctoral/post-doctoral	*n* = 3 (6%)	*n* = 2		*n* = 1
ASH total score	*m* = 355, *sd* = 93.0	*m* = 282, *sd* = 55.0	*m* = 377, *sd* = 20.1	*m* = 471, *sd* = 53.4
SXHX total score^2^	*m* = 1.7 *sd* = 0.34	*m* = 1.7 *sd* = 0.29	*m* = 1.8 *sd* = 0.23	*m* = 1.8 *sd* = 0.29

1 *Denotes number of the group pursuing next level of education full time*.

2 *No statistically significant difference between groups F(2,50) = 1.26, p = 0.293*.

### Measures

As noted above, one childhood measure of sensori-motor functioning and two adult measures, one of sensori-motor functioning and one of quality of life, were completed.

#### Childhood Measure

*OTA the Koomar Center Sensory History* (*SXHX*). The *OTA the Koomar Center Sensory History* consists of several comparable versions of a parent-report clinical sensori-motor history measure used in a sensory integration clinic to provide detailed information on the sensory processing and integration, motor (praxis) and social–emotional functioning of children and adolescents who experience sensori-motor difficulties. Various versions of the measure are appropriate for individuals from infancy to 18-years of age. Most versions included check boxes for reporting the presence of common diagnoses found to be comorbid with sensori-motor challenges (e.g., ADHD/ADD, Anxiety, Depression, Autism/Pervasive Developmental Disorder, and Learning Disabilities/Non-Verbal Learning Disorder) with an early version which only included a write-in response for reporting of diagnoses. Each version of the measure included comparable sensori-motor questions divided into the sensory areas of visual spatial, auditory, movement (vestibular), taste and smell, tactile processing, motor coordination, and a social emotional section. A 3-point Likert scale (1 = Never/Rarely, 2 = Sometimes and 3 = Often/Always) was used for all ratings except a few versions of the school-aged *SXHX*. All ratings were converted to a 3-point scale for analysis as needed. Comparability of the 3-point and 5-point scales has been examined by the authors in previous analyses. The *SXHX* is a clinical tool which has not been standardized (OTA The Koomar Center, 1981, 1993, 2004, Unpublished Measure). The school-aged *SXHX* with a 5-point response scale has strong concurrent validity with the *Sensory Profile* (Dunn, 1999) for this population with Total Score on the *SXHX* and the *Sensory Profile* having a Pearson correlation coefficient of −0.80, *p* < 0.001, individual sensory, motor, and social emotional sections have Pearson *r*’s of −0.41 to −0.81, *p*’s < 0.001 with equivalent sections on the *Sensory Profile* (May-Benson, et al., Unpublished Data). For comparison with *ASH* scores, scores on the *SXHX* were calculated for Total Score and the following section scores: Visual/Spatial Processing, Auditory/Language Processing, Movement (Vestibular), Taste/Smell, Touch, Motor Coordination, and Social/Emotional Functioning. On this measure, low scores indicate more typical sensori-motor functioning.

#### Adult Measures

*World Health Organization Quality of Life-Brief (WHOQOL-BREF).* The *WHOQOL-BREF* is a 26-question measure for adults responded to on a 5-point Likert scale based on the participants’ experiences during the previous 2 weeks. There is no total score, but scores are obtained for four domains: Physical Health, Psychological Health, Social Relationships, and Environment. There are three to seven items per domain, which are summed then scaled to be directly comparable to the *WHOQOL-100*. On this measure, high scores indicate better QOL. The *WHOQOL-BREF* has good discriminant and content validity, internal consistency and discriminant reliability (Skevington et al., 2004).

*Adult/Adolescent Sensory History*. The *ASH* (May-Benson, 2015) is a standardized 163-item self-report questionnaire for ages 13–95 years. The *ASH* is based on Ayres Sensory Integration® model of sensori-motor functioning that assesses behaviors thought to reflect sensory processing in various sensory systems, sensory modulation, sensory discrimination, praxis/postural challenges and social/emotional functioning. Items are rated on a 5-point Likert scale (1 = Never, 5 = Always). *ASH* items assess functioning in the following areas: Visual/Spatial Processing, Auditory/Language Processing, Movement (Vestibular), Taste/Smell, Touch, Proprioception, Postural Control, Motor Coordination Skills (praxis), and Social/Emotional Functioning. Results include a Total Score, individual subscores for each sensory processing and integration/motor/social area as well subscores for sensory modulation and sensory discrimination functions. Only Total Score, Modulation, Discrimination and individual sensory processing and integration/ motor/social subscores are reported here. All scores are categorized based on Typical (score range up to +1.0 SD), Mild Difficulty (score range of +1.0 to +2.0 SD), and Definite Difficulty (greater than +2.0 SD) performance. On this measure high scores indicate more dysfunction. All subscores were validated through factor and Rasch analysis of items (May-Benson, 2015). Internal consistency of the Total Score and sensory sections using Cronbach’s Coefficient Alpha was 0.80–0.97. The *ASH* has good inter-rater (*r* = 0.68 for Total score and 0.39–0.77 for subscores) and test-re-test reliability (*r* = 0.85 for Total Score and 0.74–0.87 for subscores; May-Benson, 2015).

### Data Analysis

Demographic data and level of sensori-motor functioning were summarized using means, standard deviations, frequency counts and percentages. Due to varying childhood sensory history versions and differences in numbers of items in sections, mean item scores on a 1–3 scale were used to describe participants’ childhood sensori-motor patterns. The number of scored items on the *SXHX* ranged from 26–149 (*m* = 117, *sd* = 14.4). *ASH* scores were used to describe current level of sensori-motor functioning. Independent two sample t-tests were used to compare the QOL domain scores from the study sample to the US sample from the *WHOQOL-BREF* field trial (Skevington et al., 2004). Multivariate analysis of covariance (MANCOVA) was completed to examine potential relationships between the four domains of QOL, demographic variables and participants’ childhood and current level of sensori-motor functioning. Results of this MANCOVA led to subsequent analyses being completed on the Total adult Group and on three sub-groups consisting of adults who currently report Typical sensori-motor functioning and those who currently report characteristics of Mild and Definite sensori-motor challenges. One-tailed Pearson Correlation analysis was used to further examine all relationships. As study hypotheses stated that sensori-motor characteristics indicating dysfunction would be associated with poorer QOL, correlations were expected to be negative given the scoring of the two measures. In addition, two multivariate linear regressions were also completed on the adult ASH and WHOQOL-BREF data. Analysis was conducted using SPSS v22 (IBM Corp, 2013).

## Results

### Demographics

Mean age at intake of the Total Group was 7.1 years as expected based on the demographics of the larger follow-up study, and current age was 27 years. Females were more likely to respond to the follow-up study invitation and be eligible for the current study than males. Thus, participants were slightly more likely to be female than expected (e.g., 40% female in the study sample compared to approximately 20% of individuals sent invitations to participate). While all participants had no known diagnoses as children, as adults, 51% of participants reported a diagnosis of ADD/ADHD, Anxiety and/or Depression. The Total Group reported an overall upper-middle to high SES-equivalent level as they were either still in full time education and/or well-educated (e.g., 66% received at least a bachelor’s degree and a further 22% were still in full time education). This finding was consistent with the participants’ parents’ education levels and the population seen at the private clinic when they were children. While all participants had known sensori-motor challenges as children, approximately half of the study sample reported Typical overall discrimination and modulation and motor skills as adults, while approximately a quarter each reported Mild and Definite Difficulties in adult sensori-motor patterns based on responses to the *ASH*.

As information was available for all respondents at two time periods, clinical intake as children and study participation in adulthood, direct comparisons over time are at the individual level. Participants were divided into three groups based on Total Score on the *ASH*. The *n*’s among the three groups were unequal with twice as many participants with Typical sensori-motor characteristics as those that reported Mild and Definite sensori-motor difficulties. Childhood sensori-motor scores were similar across these groups, as were all other demographics. See Table 1 for details.

Before examination of sensori-motor patterns to QOL relationships, overall performance on domains of the *WHOQOL-BREF* of the Total Group were compared to known values of the WHOQOL-BREF to examine how the current study sample compared to a known population sample. The current sample scored the same as published US data from WHO (Skevington et al., 2004) for the Physical Health and Psychological Health domains. The current Total Group scored significantly better on QOL than the WHO data for the Social Relationships [*t* (210) = 2.5, *p* = 0.014] and Environment domains [*t*(210) = 13.0, *p* < 0.001]. See Table 2 for details.

**Table 2:** Comparison of *WHOQOL-BREF* domains of adults with known childhood sensory processing challenges and published data.

WHOQOL-BREF domains	Total group	*ASH* group			WHO 2004 USA^*^
		Typical	Mild difficulties	Definite difficulties	
Physical health	*M* = 16.4 *SD* = 2.6	*m* = 17.5 *sd* = 2.4	*m* = 16.4 *sd* = 1.8	*m* = 14.6 *sd* = 2.8	*M* = 15.5 *SD* = 3.2
Psychological health	*M* = 14.2 *SD* = 2.9	*m* = 15.3 *sd* = 2.7	*m* = 13.4 *sd* = 2.6	*m* = 12.8 *sd* = 2.8	*M* = 13.8 *SD* = 3.2
Social relationships	*M* = 14.6 *SD* = 3.4	*m* = 15.9 *sd* = 3.1	*m* = 13.3 *sd* = 3.3	*m* = 13.2 *sd* = 3.5	*M* = 13.2 *SD* = 3.6
Environment	*M* = 17.0 *SD* = 2.1	*m* = 17.8 *sd* = 1.8	*m* = 16.8 *sd* = 1.9	*m* = 15.8 *sd* = 2.1	*M* = 11.7 *SD* = 2.7

* *Age and gender adjusted.*

A number of demographic variables, (i.e., age, gender, diagnoses or no diagnosis, education level), known to be related to QOL, were then included as independent variables in a MANCOVA with Total Score on the *SXHX* and Total Score on the *ASH* to determine the possible influence of these variables on the four domains of current QOL. Education level was included in the analysis as an SES proxy. Results of the MANCOVA found that, for this sample, there were no relationships between the four domains of QOL on the *WHOQOL-BREF* and the demographic variables of diagnosis, education, gender, age at completion of childhood *SXHX* or current age. Additional results will be reported below.

### Childhood Sensory Processing and Adult Quality of Life

Contrary to our hypothesis, the initial MANCOVA reported above found no significant relationship between overall childhood sensori-motor functioning and adult QOL in the Total Group when included with other demographic and adult sensori-motor variables. However, we wished to examine this relationship further, so a one-tailed Pearson Correlation (given our initial directional hypotheses) was conducted on childhood Total Score on the *SXHX* with adult *WHOQOL-BREF* domains. Overall (Total Score) sensori-motor patterns were only found to have a small relationship with Physical Health (*r* = −0.23, *p* = 0.051) that approached significance.

We then further examined the relationship in the Total Group between specific sensory systems in childhood with adult QOL with a one-tailed Pearson correlation. Of the specific sensory areas, only childhood visual processing and motor coordination (praxis) were related to aspects of adult QOL. Small significant correlations among visual processing in childhood were related to Physical Health (*r* = −0.29, *p* = 0.017) and Psychological Health (*r* = −0.27, *p* = 0.027) in adulthood. Most notably there was a small significant relationship among the motor coordination (praxis) subsection and all QOL domains of Physical Health (*r* = −0.24, *p* = 0.045), Psychological Health (*r* = −0.25, *p* = 0.034), Social Relationships (*r* = −0.26, *p* = 0.030) and Environment (*r* = −0.23, *p* = 0.046).

We then further examined whether there were different relationships within the adult *ASH* subgroups. One-tailed Pearson correlations were conducted between Total *SXHX* Score and sensory system subscores and adult QOL for each *ASH* subgroup (e.g., Typical, Mild Difficulties, Definite Difficulties). No significant relationships were found between childhood sensori-motor patterns and later QOL in any *WHOQOL-BREF* category in the Typical or Mild Difficulties Groups. Notably the correlations among these two groups were predominantly very small (in the *r* = 0.00 to −0.20 range) and were highly non-significant. Only the Definite Difficulties Group had significant relationships among sensori-motor pattern subscores on the childhood *SXHX* and adult *WHOQOL-BREF* domains.

The Definite Difficulty Group had a moderate significant relationship among overall (Total Score) sensori-motor functioning and Physical Health (*r* = −0.56, *p* = 0.018). This was consistent with the finding of the Total Group. Visual processing (*r* = −0.76, *p* = 0.001), movement (vestibular) processing (*r* = −0.48, *p* = 0.042) and tactile processing (*r* = −0.63, *p* = 0.008) also all had moderate to large significant relationships with Physical Health. Visual processing (*r = −0.54, p = 0.024*) was also significantly related to Psychological Health. Motor Coordination was not significant for this Group but trended to significance (*p* = 0.067 and 0.077, respectively) for Physical Health (*r* = −0.42) and Psychological Health (*r* = −0.41).

### Current Quality of Life and Sensori-Motor Characteristics in Adults With Known Childhood Sensori-Motor Challenges

#### Total Group

As hypothesized, the initial MANCOVA on the Total Group reported above found a strong relationship among current adult sensori-motor functioning on the Total Score of the *ASH* and current QOL domains on the *WHOQOL-BREF*, *F*(4,41) = 6.4, *p* < 0.001; *Wilk’s Λ* = 0.62, *partial* *η*^2^ = 0.38. Each of the four QOL domains was significantly related to the Total Score of the *ASH* with *F*(1,44) ranging from 9.4–17.7, all *p* ≤ 0.004.

To further examine which aspects of adult sensori-motor functioning were related to specific QOL domains for the Total Group, Pearson correlations were completed on the individual sensory systems, Modulation, Discrimination and the four *WHOQOL-BREF* domains. Results found all *ASH* sensory/motor/social subsections had moderate significant correlations with all four domains of QOL in the Total Group as hypothesized which was consistent with the initial MANCOVA on Total Score. See Table 3 for details.

**Table 3:** Results of follow-up MANOVA with Bonferroni adjustment of *WHOQOL-BREF* domains by *ASH* categories.

WHOQOL-BREF subsections	*ASH* categories	Mean difference	Std. error	Sig. level^1^	95% Confidence interval
Physical Health	Typical – Mild	1.1	0.80	0.531	−0.9–3.1
	Typical – Definite	2.8	0.78	0.002	0.9–4.8
	Mild – Definite	1.7	0.91	0.185	−0.5–4.0
Psychological Health	Typical – Mild	1.9	0.92	0.116	−0.3–4.2
	Typical – Definite	2.5	0.90	0.021	0.3–4.7
	Mild – Definite	0.6	1.0	1.00	−2.0 - 3.2
Social Relationships	Typical – Mild	2.6	1.1	0.070	−0.2–5.3
	Typical – Definite	2.7	1.1	0.049	0–5.3
	Mild - Definite	0.10	1.2	1.00	−3.0–3.2
Environment	Typical – Mild	1.0	0.65	0.382	−0.6–2.6
	Typical – Definite	2.0	0.63	0.007	0.5–3.6
	Mild – Definite	1.0	0.73	0.509	−0.8–2.8

1 *Bonferroni correction.*

To examine which aspects of adult sensori-motor functioning were most important to QOL two multivariate linear regressions were conducted. First, analysis, including only adult Modulation and Discrimination subscores on the *ASH* and *WHOQOL-BREF*, was completed. All demographic variables were excluded as they were non-significant in the first model. In this analysis, the model (intercept, Modulation and Discrimination) accounted for approximately one quarter of the variation in each QOL domain (adj *R*^2^s 0.22–0.31). Modulation was highly non-significant (*p* = 0.967) while Discrimination was statistically significant with *F*(4,47) = 3.5, *p* = 0.015; *Wilk’s Λ* = 0.77, *partial η^2^* = 0.23. Discrimination was related to all four QOL domains with *p* ≤ 0.01. The second analysis included the independent variables of individual sensori-motor functions. Again, approximately one quarter of the variability in the QOL domains was accounted for by the model (Adj *R*^2^s 0.22–0.36). This analyses found only Motor Coordination (praxis) was significantly related to QOL *F*(4,41) = 4.0, *p* = 0.007; *Wilk’s Λ* = 0.72, *partial η^2^* = 0.28. Specifically, Motor Coordination (praxis) was found to be related to the QOL domains of Psychological Health (*p* = 0.011) and Environment (*p* = 0.001).

#### ASH Subgroups

As current level of sensori-motor function, as identified by the *ASH* subgroups, had varying patterns of relationships among childhood sensori-motor patterns and QOL, we further examined current level of sensori-motor function in relation to the four domains of QOL in adults. A follow-up MANOVA with *post hoc* testing was conducted with the *ASH* subgroup (e.g., Typical, Mild Difficulties, Definite Difficulties) as the only independent variable. Statistically significant differences between *ASH* subgroups were detected *F*(8,94) = 2.4, *p* < 0.019; *Wilk’s* *Λ* = 0.69, *partial* *η*^2^ = 0.17. *Post hoc* comparisons with Bonferroni corrections confirmed a statistically significant difference in all four domains of the *WHOQOL-BREF* for the Typical and Definite Difficulties *ASH* subgroups. The Mild Difficulties subgroup was not statistically different from either of the other subgroups. Adults with Definite Difficulties in current sensori-motor patterns had the poorest QOL. Adults with Typical current sensori-motor patterns had the best QOL outcomes.

As overall sensori-motor functioning had varying relationships with *WHOQOL-BREF* by *ASH* subgroups we further examined whether specific subsections of sensory/motor/social functioning on the *ASH* (e.g., modulation, discrimination, sensory systems, motor/postural or social sections) had different relationships to the four *WHOQOL-BREF* domains by *ASH* subgroups. The assumption being that the sensory processing subsections (as expected with the Total Score) would also be inversely related to the QOL domains. One-tailed Pearson correlations were therefore conducted on the sensory/motor/social functioning subscores for the three *ASH* subgroups (Typical, Mild Difficulties, Definite Difficulties) for each of the four QOL domains. For these adult sensori-motor characteristics varying relationships among QOL domains and aspects of sensori-motor functioning emerged as they did with childhood sensori-motor characteristics and QOL.

#### Typical Group

The Typical Group demonstrated moderate significant relationships between Modulation and Discrimination and Physical Health (*r* = −0.32, *p* = 0.032 and *r* = −0.35, *p* = 0.040) and Social Relationships (*r* = −0.44, *p* = 0.012 and *r* = −0.43, *p* = 0.015) that were very similar. Discrimination was also significantly related to Psychological Health (*r* = −0.36, *p* = 0.037) where Modulation was not. Significant moderate to large relationships (*r* = −0.44 to - 0.77, *p* = 0.012 to <0.001) were found among the Motor Coordination (Praxis) and Social Emotional *ASH* subsections and all *WHOQOL-BREF* domains. Taste and Smell had moderate significant relationships with the Physical Health (*r* = −0.54, *p* = 0.002), Psychological Health (*r* = −0.43, *p* = 0.014) and Social Relationships (*r* = −0.55, *p* = 0.002) domains. Tactile processing had similar significant relationships with Physical Health (*r* = − 0.34, *p* = 0.044) and Proprioception was also similar with relationships with Physical Health (*r* = − 0.41, *p* = 0.020) and Psychological Health (*r* = −0.34, *p* = 0.043). Auditory processing had significant relationships with Psychological Health (*r* = −0.39, *p* = 0.025) and Social Relationships (*r* = −0.65, *p* < 0.001). Visual processing had a significant relationship with Social Relationships (*r* = −0.39, *p* = 0.026).

#### Definite Difficulty Group

The Definite Difficulty Group demonstrated a different pattern of significant relationships. Discrimination was significantly related to the *WHOQOL-BREF* domains of Physical Health (*r* = −0.55, *p* = 0.021) and Social Relationships (*r* = −0.51, *p* = 0.030); however, no statistically significant relationships with Modulation were found. Moderate significant relationships were found among the Social Emotional Functioning *ASH* subsection and the *WHOQOL-BREF* domains of Physical Health (*r* = −0.57. *p* = 0.016), Psychological Health (*r* = −0.48, *p* = 0.042), and Environment (*r* = −0.52, *p* = 0.028). Tactile functioning had significant relationships with Physical Health (*r* = −0.50, *p* = 0.034), Psychological Health (*r* = −0.55, *p* = 0.020) and Social Relationship (*r* = −0.63, *p* = 0.008) QOL domains. Taste and Smell had similar significant relationships with QOL domains of Physical Health (*r* = −0.46, *p* = 0.049), Psychological Health (*r* = −0.52, *p* = 0.030) and Environment (*r* = −0.50, *p* = 0.034). Movement (Vestibular) had significant relationships with Social Relationships (*r* = −0.48, *p* = 0.042) and Environment (*r* = −0.56, *p* = 0.019). Auditory processing only had a significant relationship with Physical Health (*r* = −0.52, *p* = 0.027); Postural Control only had a significant relationship with Psychological Health (*r* = −0.54, *p* = 0.023); and Motor Coordination (Praxis) only had a significant relationship with Environment (*r* = −0. 65, *p* = 0.006).

#### Mild Difficulties Group

In the Mild Difficulties Group Discrimination was only significantly related to Social Relationships (*r* = −0.53, *p* = 0.031). A moderate significant relationship was also found between Taste and Smell and the Social Relationships (*r* = −0.50, *p* = 0.039) and Environment (*r* = 0.57, *p* = 0.021) QOL domains. All other relationships were non-significant. Thus, as adults, some sensori-motor patterns may have a stronger relationship with certain areas of QOL depending on the severity of current sensori-motor characteristics. See Table 4 for details.

**Table 4:** 1-tailed Pearson correlations of adult QOL on *WHOQOL-BREF* and sensori-motor functions on *ASH* (*N* = 53).

ASH subscores	Physical health				Psychological health				Social relationships				Environment			
	Total	Typical	Mild	Definite	Total	Typical	Mild	Definite	Total	Typical	Mild	Definite	Total	Typical	Mild	Definite
Total	−0.60	−0.50	0.00	−0.55	−0.55	−0.49	−0.24	−0.55	−0.52	−0.53	−0.25	−0.49	−0.52	−0.25	−0.27	−0.56
*ASH*	*p* < 0.001	*p* = 0.004	ns	*p* = 0.020	*p* < 0.001	*p* = 0.005	ns	*p* = 0.022	*p* < 0.001	*p* = 0.003	ns	*p* = 0.038	*p* < 0.001	ns	ns	*p* = 0.019
Visual	−0.46	−0.20	0.27	−0.36	−0.37	−0.13	0.14	−0.	−0.42	−0.39	0.17	−0.29	−0.45	−0.09	−0.33	−0.32
	*p* < 0.001	ns	ns	ns	*p* = 0.003	ns	ns	ns	*p* = 0.001	*p* = 0.026	ns	ns	*p* < 0.001	ns	ns	ns
Auditory	−0.49	−0.22	0.07	−0.52	−0.41	−0.39	0.37	−0.22	−0.53	−0.65	−0.03	−0.35	−0.40	−0.21	−0.23	0.09
	*p* < 0.001	ns	ns	*p* = 0.027	*p* = 0.001	*p* = 0.025	ns	ns	*p* < 0.001	*p* < 0.001	ns	ns	*p* = 0.002	ns	ns	ns
Movement	−0.44	−0.13	−0.11	−0.27	−0.39	−0.09	−0.06	−0.34	−0.47	−0.20	−0.36	−0.48	−0.47	−0.07	−0.25	−0.56
	*p* < 0.001	ns	ns	ns	*p* = 0.002	ns	ns	ns	*p* < 0.001	ns	ns	*p* = 0.042	*p* < 0.001	ns	ns	*p* = 0.019
Taste and Smell	−0.60	−0.54	−0.18	−0.46	−0.54	−0.43	−0.21	−0.52	−0.44	−0.55	−0.50	−0.36	−0.40	−0.25	0.57	−0.50
	*p* < 0.001	*p* = 0.002	ns	*p* = 0.049	*p* < 0.001	*p* = 0.014	ns	*p* = 0.030	*p* < 0.001	*p* = 0.002	*p* = 0.039	ns	*p* = 0.002	ns	ns	*p* = 0.034
Tactile	−0.53	−0.34	0.11	−0.50	−0.48	−0.26	−0.20	−0.55	−0.47	−0.31	−0.05	−0.63	−0.41	−0.04	0.03	−0.44
	*p* < 0.001	*p* = 0.044	ns	*p* = 0.034	*p* < 0.001	ns	ns	*p* = 0.020	*p* < 0.001	ns	ns	*p* = 0.008	*p* = 0.001	ns	ns	ns
Proprioception	−0.51	−0.41	−0.35	−0.19	−0.42	−0.34	−0.24	−0.17	−0.32	−0.24	−0.15	−0.03	−0.27	−0.12	0.09	0.09
	*p* < 0.001	*p* = 0.020	ns	ns	*p* = 0.001	*p* = 0.043	ns	ns	*p* = 0.010	ns	ns	ns	*p* = 0.026	ns	ns	ns
Postural Control	−0.44	−0.30	0.05	−0.20	−0.47	−0.27	−0.19	−0.54	−0.31	−0.06	−0.10	−0.28	−0.30	0.03	0.15	−0.34
	*p* < 0.001	ns	ns	ns	*p* < 0.001	ns	ns	*p* = 0.023	*p* = 0.013	ns	ns	ns	*p* = 0.014	ns	ns	ns
Motor Coordination	−0.53	−0.57	0.12	−0.20	−0.55	−0.61	−0.06	−0.33	−0.43	−0.44	−0.08	−0.14	−0.61	−0.46	−0.34	−0.65
	*P* < 0.001	*p* = 0.001	ns	ns	*p* < 0.001	*p* < 0.001	ns	ns	*p* = 0.001	*p* = 0.012	ns	ns	*p* < 0.001	*p* = 0.009	ns	*p* = 0.006
Social Emotional	−0.66	−0.66	−0.03	−0.57	−0.67	−0.77	−0.36	−0.48	−0.53	−0.54	−0.46	−0.21	−0.55	−0.44	−0.04	−0.52
	*p* < 0.001	*p* < 0.001	ns	*p* = 0.016	*p* < 0.001	*p* < 0.001	ns	*p* = 0.042	*p* < 0.001	*p* = 0.002	ns	ns	*p* < 0.001	*p* = 0.012	ns	*p* = 0.028
Modulation	−0.49	−0.32	0.15	−0.26	−0.40	−0.24	0.41	−0.33	−0.42	−0.44	0.56	−0.28	−0.38	−0.04	0.26	−0.33
	*p* < 0.001	*p* = 0.032	ns	ns	*p* < =001	ns	ns	ns	*p* < =001	*p* = 0.012	ns	ns	*p* ≤ 003	ns	ns	ns
Discrimination	−0.58	−0.35	−0.15	−0.55	−0.52	−0.36	−0.44	−0.41	−0.54	−0.43	−0.53	−0.51	−0.50	−0.23	−0.39	−0.38
	*p* < 0.001	*p* = 0.040	ns	*p* = 0.021	*p* < 0.001	*p* = 0.037	ns	ns	*p* < 0.001	*p* = 0.015	*p* = 0.031	*p* = 0.030	*p* < 0.001	ns	ns	ns

## Discussion

The findings of this study provide new and unique insight into the importance of childhood sensori-motor functioning to adult QOL. It examined the relationship between childhood sensori-motor characteristics and later adult quality of life and the relationship between adult sensory processing and adult quality of life in adults with known childhood sensori-motor challenges.

### Key Findings

In the Total Group, visual processing in childhood had a small relationship to Physical and Psychological Health in adulthood. Motor coordination (praxis) had a small relationship to all QOL domains including Physical Health, Psychological Health, Social Relationships and Environment. Approximately, 50% of the study adults with childhood sensori-motor difficulties reported Typical adult sensori-motor functioning. Twenty-five percent each reported characteristics which reflected Mild and Definite Difficulties with sensori-motor functioning. In those adults that reported Typical functioning or Mild Difficulties in sensori-motor functioning there were no significant relationships among childhood sensori-motor challenges and adult quality of life. However, in those adults that reported Definite sensori-motor difficulties as adults, childhood characteristics in areas of visual processing, tactile processing and movement (vestibular) processing were related to the Physical Health domain of QOL. Visual processing was also significantly related to Psychological Health. Motor coordination (praxis) approached a significant relationship with Physical Health. Therefore, results suggest that childhood sensori-motor challenges in areas of visual, tactile and movement (vestibular) sensory processing and motor coordination (praxis) are related to later Physical and Psychological Health aspects of quality of life, particularly in those individuals whose childhood sensory processing challenges persist into adulthood. Most importantly, when early sensori-motor challenges are not resolved in adulthood, these adult sensori-motor challenges are further related to current QOL across all domains. Conversely, if childhood sensori-motor challenges are remediated by adulthood childhood sensori-motor patterns do not appear to relate to adult QOL.

In the Total Group of adults, current sensori-motor functioning was related to all QOL domains. Of note, Sensory Modulation and Sensory Discrimination were related to both Physical Health and Social Functioning domains of QOL while Sensory Discrimination was also related to Psychological Health. Results also found that when adults report current sensori-motor characteristics of Definite Difficulty with sensori-motor functions only Sensory Discrimination was significantly related to the Physical Health and Social QOL domains. Further examination found that in the Total Group adult Motor Coordination (praxis) and Social Emotional functioning was also significantly related to all QOL domains. Specific sensori-motor functions were significantly related to varying aspects of QOL. Tactile processing was significantly related to Physical Health. Proprioceptive processing was related to Physical and Psychological Health. Auditory processing was related to Psychological Health and Social. Taste and Smell were related to Physical Health, Psychological Health and Social. Lastly, visual processing was related to the Social QOL domain. Therefore, in adults with known sensori-motor challenges as children, adult sensori-motor characteristics are significantly related to adult QOL, particularly Physical and Psychological Health.

### Relation of Findings to the Literature

These are the first findings to examine adults with known sensori-motor challenges in childhood and the first to compare childhood sensori-motor characteristics with later QOL. Findings related to childhood sensori-motor functioning and adult QOL have not previously been addressed in the literature. This study provided new insight into this relationship. This study has also contributed to understanding of the relation of adult sensori-motor characteristics with adult QOL. Many factors can impact adult QOL, and sensori-motor skills, both in childhood and as adults, are only two factors. QOL is an important aspect of life. Sensory processing and integration challenges, specifically sensory modulation, and motor coordination difficulties have been found to be related to decreased QOL. Previous studies examined aspects of sensory processing using the sensory processing model proposed by Dunn (1999). This study examined sensori-motor functions using Ayres Sensory Integration® model. Varying sensori-motor skills were found to be related to the four QOL domains. The finding that Motor Coordination functioning in adults was significantly related to all QOL domains was consistent with studies on DCD and QOL in adults (Tal-Saban et al., 2014). Social Emotional functioning (which included questions on anxiety, depression, making friends, etc.) was also related to all QOL domains. This finding was similar to previous findings by Kinnealey et al. (2011). The relationship of specific sensory systems to current QOL was also illuminated. Along with Motor Coordination skills, Auditory processing, Taste and Smell processing and Tactile processing were found to be related to Physical and Psychological Health and Social relationships.

Some previous studies examined adults with current sensory processing challenges (Kinnealey et al., 2011) or developmental coordination disorder (Tal-Saban et al., 2014; Smits-Engelsman et al., 2018; Cleaton et al., 2021), but most have not identified specific sensory or motor characteristics in their populations. Some studies suggested that sensory hypersensitivities in some of these areas of sensory processing are related to QOL, but this study is the first to examine overall sensori-motor patterns to QOL, as well as specifically the relation of Sensory Modulation and Discrimination skills to QOL. This study found that Sensory Modulation and Discrimination accounted for approximately one quarter of the variance in each QOL domain. When each was examined, Modulation was highly non-significant while Discrimination was statistically significant and significantly related to all four QOL domains. These findings provide new information on the important role of Sensory Discrimination skills on current QOL and the relation of specific sensori-motor characteristics on QOL in adults with known childhood sensori-motor difficulties. Further, this study suggests that individuals who report typical or only mild difficulties with sensori-motor functioning as adults following childhood sensori-motor dysfunctions, are likely to experience a higher QOL than adults whose childhood sensori-motor challenges persist into adulthood.

### Limitations

While many factors may impact current QOL, an individual’s life history may also have an influence. This study found that the current study sample of adults with known childhood sensori-motor challenges based on ASI is representative of a US national sample in relation to two of four areas of QOL. The current sample had significantly higher Social Relationships and Environmental QOL than the national sample. The current sample is known to consist of individuals with a higher-than-average educational status, reflecting a higher socio-economic status, which is likely related to this finding. There were no significant relationships among QOL and other demographic variables including gender, diagnoses, age at childhood intake or adult age of questionnaire completion. Although the higher educational status of this study group may influence overall QOL, there is no evidence that this population characteristic affected the relationships among sensori-motor patterns and QOL in this sample.

These results are clearly initial findings. This sample was largely homogenous, the sample size was small and subgroup sample sizes were smaller. Multiple correlations could possibly result in some significant relationships, but the robustness of the significant relationships and the extreme non-significance of the other findings suggest that findings are worth further examination. This group consists of a larger than expected number of adult female participants compared to the known predominately male child population. It is possible that this may lead to some bias in the results or the population under examination. Lastly, due to differences in the rating scales of the childhood sensory history versions, it was necessary to convert some scores from a 5 point to a 3-point scale which could also have contributed to some statistical drift in results.

### Conclusion

In summary, this study found that when individuals with childhood sensory processing and integration with motor coordination challenges report that their sensori-motor issues are largely resolved in adulthood, those adults have a good QOL. However, adults who continue to have sensori-motor challenges from childhood have poorer QOL as adults. In these individuals, childhood visual, movement (vestibular) and tactile sensory processing, in particular, were related to later physical and psychological health. While sensory modulation functions have traditionally been evaluated in studies about adult QOL, sensory discrimination functions have not, and this study suggests that sensory discrimination and motor coordination (praxis) skills also play an important role in QOL. These findings make sense as the Physical Health domain of the *WHOQOL-BREF* asks questions about participation in work and activities, pain, energy, sleep and mobility. Individuals with sensory modulation problems, especially those with sensory hyper-responsivity, are likely to exhibit pain and sleep challenges. Also, the three areas of sensory processing identified, and specifically sensory discrimination functions are known to be related to motor coordination and praxis functions and therefore may contribute to challenges in mobility, as well as, participation in work and activities (Bundy and Lane, 2020).

The Psychological domain of the *WHOQOL-BREF* asks about areas such as positive feelings, thinking, self-esteem, negative feelings and body image. Children with patterns of sensori-motor dysfunction as identified by Ayres are known to have challenges in many of these areas. Motor coordination (praxis) challenges are known to be related to self-esteem and poor body awareness. Difficulties in tactile and vestibular sensory discrimination are known to be related to decreased motor skills and decreased self-esteem and body image as well (Bundy and Lane, 2020). Thus, the relation of our findings to these areas of QOL is reasonable. Challenges in any of these areas is likely to contribute to difficulties in participation in daily life activities from self-care activities to participation in work and leisure activities.

Much additional information is needed to fully understand the results of this study and to understand the potential relationships among sensori-motor functions and QOL. Clearly, the change in sensori-motor functioning from childhood to adulthood plays an important role as those individuals who persist in having difficulties with sensori-motor functioning as adults reported poorer QOL than those who reported their childhood sensori-motor challenges were remediated. Factors that might contribute to this change may also contribute to QOL. The amount and type of therapy received from childhood to adulthood would be important to examine including mental health and occupational therapy services. This sample was very homogenous across SES/education, diagnoses, and other demographic variables but examination of a more diverse group may yield different findings. Lastly, this study is the first known longitudinal follow-up study that has included participants that have been followed up over 15+ years later. Additional studies which follow-up children with known sensori-motor challenges as adults are needed, both retrospectively and prospectively. Understanding factors which may contribute to adult functioning for these children is needed for clinicians to provide accurate prognosis information to families and to guide intervention.

## Data Availability Statement

The raw data supporting the conclusions of this article will be made available by the authors, without undue reservation.

## Ethics Statement

The studies involving human participants were reviewed and approved by Spiral Foundation Institutional Review Board, protocol #1039. The patients/participants provided their written informed consent to participate in this study.

## Author Contributions

TM-B, OE-D, and AT contributed equally to the conceptualization of the study and writing and editing of the manuscript. AT contributed with statistical analysis. TM-B and AT contributed with data analysis and interpretation of the study data. All authors contributed to the article and approved the submitted version.

## Funding

This research was sponsored by funding from OTA The Koomar Center, Newton, MA in a fee for hire agreement with the Spiral Foundation.

## Conflict of Interest

TM-B is owner of TMB Educational Enterprises, LLC at the time of this study. She was Executive Director of the SPIRAL Foundation at the time of initial data collection for the larger study, from which data for this study was obtained. TMB Educational Enterprises LLC has no financial or commercial relationship with the funding agency that could be construed as a potential conflict of interest. As owner of TMB Educational Enterprises, LLC, TM-B received no financial or commercial benefit from this study. AT is employed by the SPIRAL Foundation and OE-D is employed by OTA The Koomar Center serving as liason to the SPIRAL Foundation. This study was funded by OTA The Koomar Center in a fee for hire arrangement with the SPIRAL Foundation. The funder contributed to initial client outreach for data collection as described in the article. The SPIRAL Foundation paid the publication fee for this study. OTA The Koomar Center was not involved in the study design, analysis, and interpretation of data or writing of this article.

## Publisher’s Note

All claims expressed in this article are solely those of the authors and do not necessarily represent those of their affiliated organizations, or those of the publisher, the editors and the reviewers. Any product that may be evaluated in this article, or claim that may be made by its manufacturer, is not guaranteed or endorsed by the publisher.

## Footnotes

^1^Dr. A. Jean Ayres, occupational therapist, developed the theory, assessment, and intervention for the field of occupational therapy practice known as Ayres Sensory Integration®. Ayres described two primary constructs of sensory integration: praxis (which depends on sensory discrimination) and sensory modulation (Bundy and Lane, 2020). Praxis, in sensory integration theory, is referred to as the ability to generate ideas for, plan and execute novel movements, and involves postural control, somatosensory processing, and bilateral integration and sequencing (Bundy and Lane, 2020). Sensory modulation involves sensory defensiveness, gravitational insecurity, aversive responses to movement, and under responsiveness to sensation (Bundy and Lane, 2020). Collectively, these sensori-motor functions are commonly referred to as sensory processing and integration and are thought to form the foundation for adaptive behavior, functional performance of daily occupations and ultimately are believed to be related to overall quality of life (QOL) in children and adults.
